# Immigrant Health Advantage? The Birth Outcomes of U.S.-Born Women in Mexico and Mexican-Origin Women in the United States

**DOI:** 10.1177/00221465251343322

**Published:** 2025-07-01

**Authors:** Erin R. Hamilton, Sara Alcay

**Affiliations:** 1 University of California, Davis, Davis, CA, USA

**Keywords:** 0.5 generation, birth outcomes, immigrants, Mexico–United States

## Abstract

The migration of Mexican immigrants and their U.S.-born children from the United States to Mexico raises questions about the health of American citizens transitioning into adulthood in Mexico. Combining data from Mexican and U.S. birth records from 2015 to 2019, we analyzed the health of 12,373 infants born to U.S.-born women delivering in Mexico and compared them to infants born to Mexican-born women in Mexico, Mexican-born women in the United States, and U.S.-born women of Mexican origin in the United States. Contrary to the immigrant health advantage in the United States, we found an infant health disadvantage for U.S.-born immigrants in Mexico. U.S.-born mothers in Mexico were younger and had lower rates of health insurance coverage, but these differences did not account for their higher likelihood of adverse infant health outcomes.

Between 2005 and 2015, more than 1 million Mexican immigrants returned from the United States to Mexico accompanied by several hundred thousand of their U.S.-born children, a shift in migration directions signaling a new era in Mexico–United States migration (Escobar Latapí and Masferrer 2022; Masferrer, Hamilton, and Denier 2019; Masferrer and Pedroza 2021; Zúñiga and Giorguli-Saucedo 2020). In the process, the large-scale migration of U.S.-born children to Mexico has created a new immigrant generation: the 0.5 generation, children who grow up in a country different from their own country of birth but the same as their parents’ country of birth (Zúñiga and Giorguli-Saucedo 2020). In 2015, the number of 0.5-generation children in Mexico was equal to the number of 1.5-generation children born in Mexico living in the United States: Both populations counted over half a million (Masferrer et al. 2019; Urban Institute 2021). Together, they made up a binational population of 1 million child immigrants in North America.

Between 2015 and 2020, return migration to Mexico from the United States slowed, the population of U.S.-born children living in Mexico stabilized in size, and the 0.5-generation cohort aged into adolescence and young adulthood (Masferrer and Pedroza 2021). Researchers have begun to study the well-being of this population of young American citizens living in Mexico. Studies have found that U.S.-born children in Mexico experience greater disadvantage than Mexican-born children in schooling and access to health care (Amuedo-Dorantes and Juárez 2022; Borja et al. 2021; Giorguli-Saucedo et al. 2021; Jacobo-Suárez 2017; Zúñiga and Giorguli-Saucedo 2020). The educational and health care barriers faced by U.S.-born immigrants in Mexico undoubtedly undermine their population health, but heretofore, no study has examined the health of the 0.5 generation in Mexico.

We study the population health of U.S.-born migrants in Mexico as they transition into adulthood by focusing on the health of their newborns. As the 0.5-generation cohort ages into adulthood, some have begun to form their own families there. Between 2015 and 2019, Mexico recorded more than 12,000 births in Mexico to U.S.-born women, one-third of whom were teenaged (Dirección General de Información en Salud 2024).^1^ Infants born to U.S.-born women in Mexico have the legal right to U.S. citizenship by their birth to U.S. citizens. Their generational status vis-à-vis immigration to the United States will be determined by their future migration trajectories. We examine infant birthweight, a standard population health indicator that reflects the health of the mother and sets the stage for the life course health of the child (Hughes, Black, and Katz 2017; Urquia, Sørbye, and Wanigaratne 2016).

In examining the health of infants born to U.S. immigrants in Mexico, we build on research on Mexican immigrant health in the United States, which has documented comparatively good health of infants born to Mexican immigrants in the United States (Fuentes-Afflick, Hessol, and Pérez-Stable 1999; Hummer et al. 1999, 2007; Markides and Coreil 1986; Riosmena, Wong, and Palloni 2013). The so-called “immigrant health advantage” may be due to immigrant selectivity: who immigrates to the United States, who stays there, and who returns to Mexico (Diaz, Koning, and Martinez-Donate 2016; Feliciano 2020; Ichou and Wallace 2019). Selectivity is assessed by comparing migrants to those who could migrate but do not (i.e., nonmigrants in the country of origin; Guendelman et al. 2015). In the case of Mexico-U.S. migration, selectivity operates in both directions, into and out of the United States. Binational data on migrants and nonmigrants in both countries are therefore necessary to assess selectivity.

Binational data and analyses are also essential because the direction of migration between Mexico and the United States has shifted. The large-scale (return) migration of adults and their children from the United States to Mexico has created an important population of U.S.-born immigrants in Mexico (Masferrer and Pedroza 2021; Zúñiga and Giorguli-Saucedo 2020). In this context, binational data and analysis are imperative for a full understanding of Mexican immigrant health.

In this study, we provide the first analysis of the health of the 0.5 generation in Mexico in a binational context, which allows us to examine the roles of immigrant selectivity, educational attainment, and health care usage of migrants in both countries. Specifically, we combined Mexican and U.S. birth records from 2015 to 2019, when the 0.5 generation in Mexico aged into reproductive ages (Masferrer et al. 2019; Zúñiga and Giorguli-Saucedo 2020). Using these data, we estimated and compared the rates of low and very low birthweight for four groups of births defined by the country of birth (Mexico vs. the United States) and the country of the mother's birth (Mexico vs. the United States): U.S.-born delivering in Mexico compared to Mexican-born in Mexico, Mexican-born in the United States, and U.S.-born of Mexican origin in the United States. Before describing our data, methods, and findings, we review the existing literature on the immigrant health advantage and the 0.5 generation in Mexico.

## Background

### The Immigrant Health Advantage

Mexican immigrant women in the United States have better birth outcomes than U.S.-born women of Mexican background and similar birth outcomes to U.S.-born White women, patterns consistent with phenomena called the “immigrant health advantage” and the “epidemiologic (or Hispanic) paradox” (Fuentes-Afflick et al. 1999; Guendelman et al. 2015; Hummer et al. 2007; Markides and Coreil 1986; Riosmena et al. 2013). The unexpected pattern of comparatively good birth outcomes among a socioeconomically disadvantaged immigrant group could be due to immigrant selectivity and/or the health behaviors and social support of immigrants (Guendelman et al. 2015; Riosmena et al. 2013; Riosmena, Kuhn, and Jochem 2017). The selection of less healthy migrants into return migration to their origin country may also explain the good health of immigrants who remain in the United States (Arenas et al. 2015; Diaz et al. 2016).

The same pattern of an immigrant health advantage may not be observed among U.S.-born women in Mexico for four reasons. First, children who migrate to accompany their parents are not theoretically subject to the same processes of immigrant selectivity as adults (Feliciano 2020). Second, the 0.5 generation was born to immigrant parents in the United States. There is evidence that the immigrant health advantage fades over time and across generations, even in early childhood among the second generation (Hamilton, Teitler, and Reichman 2011; Schmeer 2019). Third, the 0.5 generation consists of the children of return migrants, who may be negatively selected on health (Arenas et al. 2015; Diaz et al. 2016). A fourth reason relates to the social disadvantages that the 0.5 generation faces in Mexico, which we describe in the next section.

Each of these reasons assumes that most U.S.-born women delivering in Mexico in our period of study, between 2015 and 2019, migrated to Mexico as children to accompany their immigrant parents returning from the United States to Mexico. To be clear, we do not observe the ancestry or timing (or age) of migration of U.S.-born women delivering in Mexico, but we assume that a large portion is the 0.5 generation. In the first stage of our analysis, we show evidence consistent with this assumption.

### The 0.5 Generation

Victor Zúñiga and Silvia Giorguli-Saucedo (2020) defined U.S.-born children in Mexico as the 0.5 generation. The large majority of 0.5-generation children immigrated to Mexico with return-migrant parents, that is, Mexican immigrants to the United States who had children there and later returned to Mexico with their children (Masferrer et al. 2019). A smaller number do not live with parents but with grandparents. Between 2005 and 2015, the population of 0.5 generation children doubled to more than half a million; after 2015, the population stabilized and aged, beginning the transition to adulthood (Masferrer et al. 2019; Masferrer and Pedroza 2021; Zúñiga and Giorguli-Saucedo 2020).

Although U.S.-born citizens in Mexico have, by jus soli, U.S. citizenship, their legal status does not translate into social advantages in Mexico as it is thought to in the United States. Multiple studies have documented the social disadvantages faced by U.S.-born children in Mexico. This disadvantage takes three forms: first, barriers to labor market entry and socioeconomic mobility faced by return migrant parents (Denier and Masferrer 2019; Mora-Rivera, Llamas-Huitron, and Cruz-Salas 2023; Parrado and Gutierrez 2016); second, legal and institutional barriers to formal educational and health care institutions faced by the children (Jacobo-Suárez 2017; Medina and Menjívar 2015; Sánchez Garcia and Hamann 2017; Vargas Valle and Ortiz Rangel 2019); and third, social stigma related to assumptions of criminality and deportation upon migration to Mexico following time spent in the United States (Anderson 2015; Schuster and Majidi 2015; Zúñiga and Hamann 2015, 2020). By undermining socioeconomic status, educational attainment, access to health care, and social integration, these conditions arguably also undermine the health of U.S.-born immigrants in Mexico and the health of their infants.

No study has examined the health of U.S.-born children in Mexico because their numbers are too small in most population health data sources. In this study, we take advantage of the recording of the mother’s place of birth on Mexican birth records and the aging of the 0.5 generation into childbearing years to examine the health of the infants born to U.S.-born women in Mexico between 2015 and 2019. We describe our study steps next.

### The Current Study

We harmonized birth records from the United States and Mexico between 2015 and 2019, compiling a census of all births occurring in the two countries in this period. We compared the health of infants born to U.S.-born mothers in Mexico to three other groups of migrants and nonmigrants, as defined by place of birth of the mother and location of delivery of the focal infant: Mexican-born mothers delivering in Mexico (i.e., the nonmigrant counterpart in Mexico), U.S.-born women of Mexican origin delivering in the United States (the nonmigrant counterpart in the United States), and Mexican-born women delivering in the United States (the immigrant counterpart in the United States).

We compared the four groups of mothers along sociodemographic characteristics recorded in the birth records, including age, parity, education, marital status, and prenatal care usage, to describe group differences in key social determinants of infant health (Klawetter 2014; Reno and Hyder 2018). Additionally, we assessed differences in group characteristics to evaluate our assumption that the population of U.S.-born women delivering in Mexico in this period is largely comprised of the 0.5 generation. In particular, we assessed the similarity in their educational and marital profiles with the other three groups under the assumption that these population distributions reflect local norms and opportunity structures.

Processes of selectivity into (return) migration coupled with the social disadvantages faced by U.S.-born children in Mexico, as described previously, led us to expect a health disadvantage in the population of U.S.-born women delivering in Mexico in comparison to Mexican-born women delivering in Mexico and to Mexican-born and U.S.-born women delivering in the United States. We used multivariable regression models to determine to what extent measured differences between women account for differences in infant health outcomes. To account for imbalanced age structures between groups, we then matched births by mother’s age and estimated age-stratified models. In comparing births to teenage immigrant mothers in the United States and Mexico, we assessed whether group differences may result from child migration. Because U.S.-born mothers face barriers to health care in Mexico, we then focused on births in Mexico, which allows us to incorporate a larger and more detailed set of health care characteristics. We describe our methods in more detail next.

## Data and Methods

### Data and Sample

We combined and analyzed birth records from Mexico and the United States from 2015 to 2019. We focused on births between 2015 and 2019 to maximize the number of births to the 0.5 generation of U.S.-born people who immigrated to Mexico as children. To be clear, neither the timing nor the age of migration of mothers is recorded in either system of birth records. However, we know that the mass migration of the 0.5 generation to Mexico occurred between 2005 and 2015 and that the 0.5 generation aged thereafter (Masferrer et al. 2019; Zúñiga and Giorguli-Saucedo 2020). In 2015, there were 550,000 U.S.-born children (under age 18) living in Mexico, representing about 60% of all U.S.-born people in Mexico; one-third of U.S.-born children (about 144,000) were already in their childbearing years (12–17 years old), and another 40% aged into their childbearing years by 2019 (Giorguli-Saucedo et al. 2021). We are unable to examine births to U.S.-born women in Mexico after 2019 because that is the last year that the records report the U.S. birth of mothers; after 2019, only foreign birth (but not country) is reported. Our focus on births between 2015 and 2019 therefore selects on early childbearing-age births among the U.S.-born in Mexico.

Mexican birth records are collected and made publicly available by the Mexican National Secretary of Health (*Secretaría de Salud*). U.S. birth records are collected by the U.S. National Vital Statistics System and made publicly available by the U.S. National Center for Health Statistics (National Center for Health Statistics 2023). The two data sets compiled a census of births occurring in Mexico and the United States between 2015 and 2019, with information regarding birth outcomes, mothers’ sociodemographic background, and mothers’ use of health care. In the Mexican birth records, we limited the analysis to births by women born in Mexico or the United States. In the U.S. birth records, we limited the analysis to women of Mexican origin. We limited both samples to singleton births born between 22 and 44 weeks of gestation and women between the ages of 10 and 49. The final analytic sample included 9,452,010 births in Mexico and 1,990,159 births in the United States.

U.S. birth records reflect the collection and recording of information on U.S. birth certificates, which confer U.S. citizenship and access to other documentation, such as passports, to infants born in the United States. The U.S. birth records include a nearly complete registration of all births occurring in the United States. In Mexico, there are two systems, one by the Civil Registry and one by the Secretary of Health. The Mexican birth certificate granted by the Civil Registry is the official document that certifies the citizenship and the name of a newborn in the country; the Secretary of Health collects medical and sociodemographic information about the infant and the mother. Although the Civil Registry document confers citizenship in Mexico, either document can be used to obtain a U.S. Consular Report of Birth Abroad, which is then used to certify U.S. citizenship and obtain a U.S. passport. Because we were interested in health, we used birth records data from the Secretary of Health.

Not all births in Mexico are registered by the Mexican Secretary of Health; rural and nonhospital deliveries are less likely to be registered (Mier y Terán Rocha and García Guerrero 2019). The number of births registered in the National Birth Records represented 96% of the total births that received a birth certificate through age four from the Civil Registry.

Several previous studies combine data sources across countries, including analyses of Mexican and U.S. birth records (e.g., Guendelman et al. 2015; McDonald et al. 2013), birth records from other countries (e.g., Guendelman et al. 1999), Mexican and U.S. survey data (e.g., Bostean 2013; Riosmena and Dennis 2012b; Riosmena et al. 2013), and survey data from other countries (e.g., Martinson, Tienda, and Teitler 2017). To achieve data harmonization, we first ensured that data collection adhered to comparable parameters (Cheng et al. 2024; Doiron et al. 2013). In both cases, birth records were reported by national health institutions, which collected data within the same time frame and applied identical inclusion criteria. We then analyzed the survey questions used to collect information about the births and the definitions, codes, and categorization schemes employed in each country. For the binational analysis, we retained only those variables that were measured in the same units or had categories that could be meaningfully compared. Next, we constructed a unified data set based on newly defined parameters.

### Measures and Analysis

We conducted two separate analyses, the first with the binational data and the second with births in Mexico. For the binational analysis, we analyzed two birthweight cutoffs: very low birthweight (< 1,500 grams) and low birthweight (< 2,500 grams; Wingate, Epstein, and Bello 2017). In the binational analysis, we focused on birthweight because of its comparability between the U.S. and Mexican data sets. Low birthweight is one of the most commonly used indicators to assess health inequalities across populations, including across countries, because its measurement is standardized and does not require specialized medical equipment. Although not all births below 2,500 grams experience health problems, low birthweight is strongly related to adverse health outcomes in infancy and childhood (Hughes et al. 2017; Urquia et al. 2016). In the binational analysis, we did not use other health indicators, such as preterm birth, because the measurement of gestational age differs in the two countries. In the analysis of births in Mexico, we included preterm birth (<36 weeks gestation).

We categorized women according to their nationality, ethnic origin, and country of delivery. Using these indicators, we created a four-category measure of migration generation: birth in Mexico to a Mexican-born woman, birth in Mexico to a U.S.-born woman (0.5 generation), birth in the United States to a Mexican-born woman (first generation), and birth in the United States to a U.S.-born woman of Mexican origin (second+ generation). The Mexican birth records include information regarding the mother’s country of birth but not ancestry, ethnicity, or race. We assumed that most U.S.-born women in Mexico are of Mexican ancestry, but some may be non-Mexican, U.S.-born people (of other ethnic and racial backgrounds) living in Mexico. The sociodemographic profile of U.S.-born people giving birth in Mexico in this period was consistent with our assumption that most are of Mexican ancestry and migrated as children (as shown in the results). It is also important to note that because we did not observe the timing or age of migration, the second+ generation of U.S.-born women of Mexican origin delivering in the United States may include some former 0.5-generation women if they migrated to Mexico and later returned to the United States prior to the focal delivery.

The nature of data collection and differences between the health care payment and delivery systems in Mexico and the United States limited the measures we could reliably compare across countries. In the binational analysis, we controlled for the following characteristics of women: age in five-year brackets, parity (first-order birth, second-order birth, or third-or-higher-order birth), marital status (formally married vs. unmarried, which includes consensual unions), educational level (highest level of schooling is junior high school or less, some high school, completed high school, or some college or more), and number of prenatal health care visits. For these variables, the harmonization process involved reducing the categories to the lowest common denominator for marital status and establishing equivalence in years of schooling for educational attainment. After comparing groups of women on these characteristics, we estimated nested multivariable regression models to assess whether group differences in birthweight change when controlling for measured differences between women. In addition to regression estimates, we present predicted rates of low birthweight. Because the educational gradient in health varies across countries and immigrant groups, we tested separate models incorporating an interaction between maternal education and generational groups (Buttenheim et al. 2010; Goldman et al. 2006; Riosmena and Dennis 2012a, 2012b).

Age emerges as an important differentiating factor among groups. To test whether the young age distribution of U.S.-born women in Mexico owes to population composition, as we would suspect if they are the 0.5 generation, or to high teenage fertility, we calculated age-specific fertility rates using counts of births by mother’s age in the 2015 birth records, expressed per woman in each age group. We obtained population counts of U.S.-born and Mexican-born women in Mexico from the 2015 Mexican Inter-censal Survey.

We subsequently matched women by age and ran age-stratified models on births to women ages 17 or younger, 18 to 29, and 30 or older. Age stratification allowed us to assess whether group differences were observed for all age groups and produced less biased estimates, similar to other forms of statistical matching (Benedetto et al. 2018). Because all other characteristics of mothers were measured after migration, we could not use propensity scores to achieve a more balanced sample or account for selection into migration. Our analysis of births to women ages 17 or younger also enabled us to focus on migration before legal adulthood, which is defined as age 18 in Mexico and the United States.

In the second part of the analysis, we limited the analysis to the Mexican birth records, which provided greater flexibility in measurement and allowed us to address inequalities in health care specific to the Mexican context. In the analysis of Mexican births, we controlled for a greater number of mothers’ marital status categories than were available in the U.S. data (married, single, and consensual union) and education categories that included groupings that were not relevant in the U.S. data (less than junior high school, which accounts for 20% of the Mexican sample; some or completed junior high school [*secundaria*]; some or completed high school [*preparatoria*]; some or completed college or higher). We also controlled for mothers’ employment (employed and unemployed), which was not recorded in the U.S. data. Additionally, we included variables related to access to health care: type of medical insurance (none, public work-based, public-universal, or other); doctor delivery, which includes physicians, obstetricians, medical residents, and other specialists, versus other delivery, which includes deliveries by nurses, midwives, or others; and place of delivery (nonmedical facility, work-based public hospital, universal care public hospital vs. private hospital). Medical insurance and place of delivery reflect the work-based division in health care in Mexico. Formal employment in the public sector grants access to insurance and hospitals through the Mexican health care programs known by their acronyms ISSSTE, PEMEX, SEDENA, and SEMAR; employment in the private sector grants access to the health care program IMSS.^2^ The second major health care system in Mexico during this period was *Seguro Popular*, a universal public health care system that offered coverage to people without formal employment. The Mexican government overhauled the *Seguro Popular* program in 2020, and it is now known as IMSS *Bienestar*.

## Results

### Binational Analysis

Table 1 shows the unadjusted means and percentage distributions of each variable in the binational analysis, comparing the four groups of births defined by the country of birth and the mother’s birthplace. We did not include statistical tests because we use a census of births (not subject to sampling) and because the cell sizes are large. We focus instead on substantive differences.

**Table 1.** Characteristics of Births and Mothers by Location of Birth and Birthplace and Ethnicity-Race of the Mother among 11,442,169 Births in Mexico and the United States from 2015 to 2019 (%).

	Births in Mexico		Births in the United States	
	Mexican-Born Mother	U.S.-Born Mother	Mexican-Born Mother	U.S.-Born Mexican Origin Mother
Low birthweight	5.3	6.4	5.3	6.1
Very low birthweight	.5	1.0	.8	.9
Male	51.0	50.9	50.9	51.0
Birth history				
First pregnancy	36.2	47.4	20.9	34.1
Second pregnancy	30.9	27.7	23.7	27.1
Third + pregnancy	32.9	24.9	55.4	38.8
Age				
10–14	.5	1.8	.0	.2
15–19	18.4	32.9	4.5	12.4
20–24	29.5	29.6	18.4	32.7
25–29	25.1	18.1	27.7	28.8
30–34	16.6	10.5	27.5	17.2
35–39	7.9	5.7	16.8	7.4
40–44	1.8	1.2	4.8	1.3
45–49	.1	.1	.2	.1
Married	36.5	34.1	55.3	44.6
Education				
Less than high school	53.0	43.6	17.8	1.7
Some high school	6.7	11.5	26.0	17.7
High school	23.0	22.3	33.1	35.1
Some college or more	17.2	22.6	23.2	45.5
Mean prenatal checkups	7.5	7.7	10.5	10.8
Number of births	9,439,637	12,373	914,498	1,075,661

*Source:* Mexican and U.S. birth records, 2015–2019.

Between 2015 and 2019, the rates of very low birthweight and low birthweight were higher among U.S.-born women than among Mexican-born women regardless of the migrant status of the mother. Among births to U.S.-born women in Mexico, 6.4% were low birthweight, and among U.S.-born women of Mexican origin in the United States, 6.1% of births were low birthweight. This compares to 5.3% of births among Mexican-born women in Mexico and Mexican-born women in the United States. The pattern for very low birthweight is similar except that there is a higher rate of very low birthweight among Mexican-born mothers in the United States as compared to in Mexico (.8% vs. .5%).

The four groups of women had different age structures and birth histories, with U.S.-born women in Mexico notably younger and more likely to be first-time mothers. Almost half (47.%) of births to U.S.-born women in Mexico are the first pregnancy, compared to about a third (36.2%) of Mexican-born women in Mexico and one-fifth (20.9%) of Mexican immigrant women in the United States. Over one-third (34.7%) of U.S.-born women who gave birth in Mexico between 2015 and 2019 were under 20 years old, compared to 18.9% of Mexican-born women in Mexico, only 4.5% of Mexican-born women in the United States, and 12.6% of U.S.-born women of Mexican origin in the United States.

**Figure 1.**
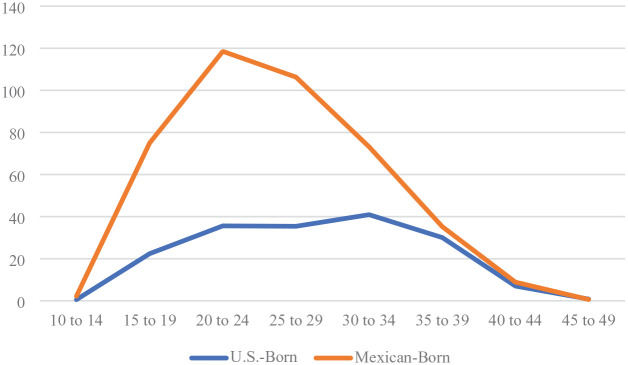
Age-Specific Fertility Rates of U.S.-Born and Mexican-Born Women in Mexico in 2015. *Source:* Mexican birth records, 2015, and Mexican Inter-censal Survey, 2015. *Note:* N = 2,095,222 births; N = 38,057,030 women.

The young age profile of U.S.-born women in Mexico is consistent with our assumption that most U.S.-born women delivering in Mexico between 2015 and 2019 migrated to Mexico as the children of Mexican immigrants returning to Mexico from the United States in the 2005 to 2015 period. To test whether the young age distribution of U.S.-born women is not a result of higher teenage fertility among this group, we calculated age-specific fertility rates (ASFRs) for the two groups of women in Mexico. Figure 1 shows that U.S.-born women had lower ASFRs than Mexican-born women, including among teenagers, in 2015. The age structure of U.S.-born delivering people in the Mexican birth records is due to the young age composition of U.S.-born women in Mexico, not high fertility.

The U.S.-born ASFRs are very low, producing a total fertility rate of .9 births per U.S.-born woman in Mexico in 2015, compared to an estimated 2.1 births per Mexican-born woman. This could be due to tempo distortion on period fertility estimates if the population of U.S.-born women in Mexico in 2015 was postponing childbearing, such as for educational purposes or in anticipation of return migration to the United States.

The marital status and educational profiles of U.S.-born women in Mexico also support our assumption that these women belong to the 0.5 generation of children who accompanied return migrant parents. U.S.-born women delivering in Mexico are more similar to Mexican-born women in Mexico than to U.S.-born women in the United States in terms of education and marital status. The percentage of women in the data set who were formally married in Mexico was similar for U.S. and Mexican-born mothers: 34.1% of U.S.-born women and 36.5% of Mexican-born women in Mexico were formally married. These profiles are different from women delivering in the United States: 55.3% of Mexican immigrants and 44.6% of U.S.-born women of Mexican origin were formally married.

The educational profiles of women differ starkly between the two countries in a pattern that further supports our assumption that U.S.-born women delivering in Mexico in this period are largely comprised of the 0.5 generation. In Mexico, the most common educational level among delivering people is less than high school, whereas this level of educational attainment is uncommon (17.8%) among Mexican immigrants delivering in the United States and rare (1.7%) among U.S.-born women of Mexican origin delivering in the United States. For reference, 1.2% of U.S.-born White people delivering in the United States had this level of education in the same period (not shown). If U.S.-born people delivering in Mexico had completed their education in the United States, we would expect them to look more similar to U.S.-born women of Mexican origin in the United States; the fact that their educational profile is more similar to Mexican-born women in Mexico suggests that these women were child migrants who completed their education in Mexico. Mothers in Mexico received on average 7.5 to 7.8 prenatal care visits, 3 fewer than women delivering in the United States. This level of prenatal care is similar for U.S.-born and Mexican-born mothers in Mexico, although later, we show that U.S.-born mothers in Mexico had lower levels of health insurance coverage than Mexican-born mothers in Mexico.

Table 2 shows coefficients from linear probability models of low and very low birthweight regressed on women’s generational group and the characteristics of the birth and mother. Model 1 includes infant sex, mother’s age, and parity, and Model 2 adds the mother’s education, marital status, and prenatal care. Not shown but included are year fixed effects. The coefficients for the mother’s generational group are interpreted as the difference in the probability of low (very low) birthweight between each group and Mexican-born women in Mexico, adjusted for the other variables in the model. Model 1 shows that U.S.-born women in Mexico have a .01 (1 percentage point) higher probability of low birthweight than Mexican-born women in Mexico even after controlling for the differences in their age profiles and birth histories. The other group differences noted in Table 1 also remain in Model 1.

**Table 2.** Linear Probability Regression Coefficients of Low and Very Low Birthweight among 11,442,169 Births to Mexican-Origin Mothers in Mexico and the United States, 2015–2019.

	Low Birthweight		Very Low Birthweight	
	(1)	(2)	(1)	(2)
	Coefficient (*SE*)	Coefficient (*SE*)	Coefficient (*SE*)	Coefficient (*SE*)
Group (reference = Mexican-born in Mexico)				
U.S.-born in Mexico	.010*** (.002)	.010*** (.002)	.005*** (.001)	.005*** (.001)
Mexican-born in U.S.	–.001*** (.000)	.008*** (.000)	.002*** (.000)	.005*** (.000)
U.S.-born Mexican origin in U.S.	.009*** (.000)	.019*** (.000)	.004*** (.000)	.007*** (.000)
Mother’s age (reference = 20–24 years)				
10–14	.025*** (.001)	.021*** (.001)	.000*** (.000)	.003*** (.000)
15–19	.006*** (.000)	.003*** (.000)	.000*** (.000)	–.000*** (.000)
25–29	–.000 (.000)	.002*** (.000)	.001*** (.000)	.001*** (.000)
30–34	.005*** (.000)	.010*** (.000)	.002*** (.000)	.003*** (.000)
35–39	.016*** (.000)	.021*** (.000)	.004*** (.000)	.006*** (.000)
40–44	.032*** (.001)	.037*** (.001)	.007*** (.000)	.008*** (.000)
45–49	.058*** (.002)	.062*** (.002)	.010*** (.001)	.011*** (.001)
Birth history (reference = first pregnancy)				
Second	–.008*** (.000)	–.009*** (.000)	–.001*** (.000)	–.002*** (.000)
Third+	–.006*** (.000)	–.009*** (.000)	–.001*** (.000)	–.002*** (.000)
Infant male sex	.002*** (.000)	.002*** (.000)	.001*** (.000)	.001 (.000)
Married		–.001*** (.001)		.000 (.000)
Education (reference = ≤ junior high school)				
Some high school		.007*** (.000)		.002*** (.000)
High school		.004*** (.000)		.002*** (.000)
Some college or more		.006*** (.000)		.002*** (.000)
Prenatal visits		–.004*** (.000)		–.001 (.000)
Constant	.049*** (.000)	.078*** (.000)	.004*** (.000)	.014*** (.000)
*R* ^2^	.0013	.0051	.0006	.0040
*F*	898.72	2,653.25	427.60	2,083.53
Number of observations	11,441,253	11,441,253	11,441,253	11,441,253

*Source:* Mexican and U.S. birth records 2015–2019.

*** *p* < .001.

Model 2 shows that controlling for group differences in education, marital status, and prenatal care does not change the coefficients for U.S.-born women in Mexico. The findings suggest that group differences in these characteristics do not account for the higher rates of low (very low) birthweight among U.S.-born immigrants in Mexico compared to Mexican-born women in Mexico.

**Figure 2.**
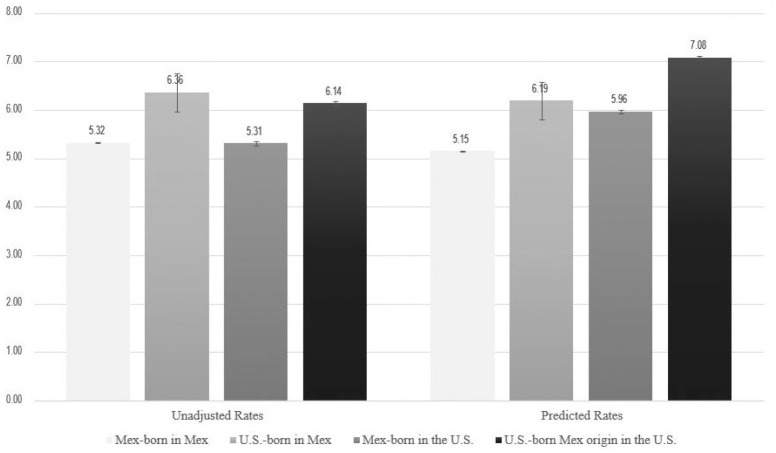
Unadjusted and Predicted Rates of Low Birthweight among 11,442,169 Births to Mexican-Origin Mothers Delivering in Mexico and the United States between 2015 and 2019, by Mother’s Country of Birth and Country of Delivery. *Source:* Mexican and U.S. birth records, 2015–2019. *Note:* Predicted rates are calculated from the estimates in Table 2, Model 2. Mex = Mexican.

Although the coefficients for U.S.-born women in Mexico do not change from Model 1 to Model 2, the coefficients for women in the United States increase across models, which means that if Mexican-origin women in the United States had similar educational, marital status, and prenatal care profiles as Mexican-born women in Mexico, they would have higher rates of low (very low) birthweight. Figure 2 presents this result visually: The unadjusted rates of low birthweight are shown on the left, and the predicted rates of low birthweight using the coefficients from Model 2 in Table 2 are on the right. Adjusted for group differences in education, marital status, and prenatal care, the predicted rate of low birthweight among U.S.-born women of Mexican origin in the United States is 7.1 percent; this is the generational group that U.S.-born women in Mexico would belong to, assuming their Mexican background, had they not migrated to Mexico. The change in the relative rates of low birthweight from the unadjusted model to Model 2 shows that the rate of low birthweight would be nearly a percentage point lower among births to U.S.-born women in Mexico compared to U.S.-born women in the United States if they had the same educational, marital, and prenatal care distributions.

We note that education has a positive association with low (very low) birthweight, meaning that women at higher levels of education have a higher rate of these adverse infant health outcomes net of other characteristics in the model. This result is driven by a positive association between education and low (very low) birthweight among both groups of women in Mexico, a finding that has been reported in other studies (Ancira-Moreno et al. 2021; Frank 2005; Frank et al. 2004; Rebollar, Esquivel, and Gómez 2010). In results not shown, we allowed the association between education and the birthweight outcomes to vary by generation group, and we observed negative coefficients on the interactions for women delivering in the United States (results available on request). For Mexican immigrant women in the United States, the association between education and the probability of low birthweight is essentially flat, a finding consistent with other research showing that the socioeconomic gradient in health is weaker for Mexican immigrants (Acevedo-Garcia, Soobader, and Berkman 2007; Goldman et al. 2006). The unexpectedly flat social gradient in infant (and other) health outcomes is an essential component of the Hispanic or epidemiologic paradox, which helps account for the better than expected health outcomes of Mexican immigrant women and the unraveling of the paradox over time (Buttenheim et al. 2010; Goldman et al. 2006; Riosmena and Dennis 2012a, 2012b). Indeed, in our data, among U.S.-born women of Mexican origin in the United States, the probability of low (very low) birthweight decreases with education. However, accounting for group differences in this association does not change the pattern of group differences in low and very low birthweight.

Our focal group of interest—U.S.-born women in Mexico—has a very different age profile than the other groups. In Table 3, we present coefficients from the same two regression models run separately by the age of the mother. There are two important findings in Table 3. First, the difference in low birthweight between U.S.-born and Mexican-born women in Mexico is positive for all age groups (ranging between .002 and .014). However, the differences are greater among younger women.

**Table 3.** Linear Probability Regression Coefficients and Predicted Probability of Low Birthweight among Births to Mexican-Origin Mothers in Mexico and the United States, 2015–2019, Stratified by Maternal Age.

	Linear Regression Coefficients		Predicted Probability	
	(1)	(2)	(1)	(2)
	Coefficient (*SE*)	Coefficient (*SE*)	% (95% CI)	% (95% CI)
<18				
Mexican-born in Mexico	(Reference)	(Reference)	6.2 (6.1–6.2)	6.1 (6.0–6.1)
U.S.-born in Mexico	.014** (.005)	.014** (.005)	7.6 (6.6–8.5)	7.5 (6.6–8.5)
Mexican-born in U.S.	.005* (.002)	.015*** (.002)	6.7 (6.3–7.1)	7.6 (7.2–8.1)
U.S.-born Mexican origin in U.S.	.017*** (.001)	.030*** (.001)	7.9 (7.6–8.1)	9.1 (8.9–9.4)
*N* = 459,803				
18–29				
Mexican-born in Mexico	(Reference)	(Reference)	5.0 (4.9–5.0)	4.8 (4.8–4.8)
U.S.-born in Mexico	.010*** (.002)	.011*** (.002)	6.0 (5.5–6.5)	5.9 (5.4–6.3)
Mexican-born in U.S.	–.000 (.003)	.009*** (.000)	5.0 (4.9–5.0)	5.7 (5.7–5.7)
U.S.-born Mexican origin in U.S.	.009*** (.000)	.020*** (.000)	5.9 (5.8–5.9)	6.8 (6.7–6.8)
*N* = 7,350,331				
≥30				
Mexican-born in Mexico	(Reference)	(Reference)	6.0 (6.0–6.1)	5.8 (5.8–5.9)
U.S.-born in Mexico	.002*** (.006)	.003 (.006)	6.2 (5.1–7.3)	6.2 (5.1–7.3)
Mexican-born in U.S.	−.001** (.000)	.007*** (.000)	5.8 (5.8–6.0)	6.5 (6.4–6.6)
U.S.-born Mexican origin in U.S.	.008*** (.001)	.016*** (.001)	6.8 (6.7–6.9)	7.5 (7.4–7.6)
*N* = 2,744,012				

*Source:* Mexican and U.S. birth records, 2015–2019.

*Note:* Model 1 controls for maternal age, parity, and infant sex. Model 2 additionally controls for maternal marital status, education, and prenatal care visits. CI = confidence interval.

* *p* < .05, **p < .01, ***p < .001.

Second, the results for births to women ages <18 provide a test of the role of child migration by limiting the sample of immigrant mothers to those who migrated before the age of legal adulthood. If child migration accounted for the higher rate of low birthweight among the U.S.-born women in Mexico, compared to their immigrant counterparts in the United States, then we would expect to see the difference between these two groups decrease in the analysis of births to mothers under age 18, who share a similar life course timing of migration (i.e., before adulthood). Table 3 shows that the rates of low birthweight are higher among mothers under age 18 than they are among older women, but we do not see a disproportionate change in the coefficient for first-generation (or 1.5-generation) Mexican-born women in the United States. This gives some evidence against the idea that child migration may account for the elevated rates of low birthweight among the 0.5 generation in Mexico.

### Analysis of Births in Mexico

We now turn to an analysis focused only on births in Mexico, in which we take advantage of the greater number of variables available in the Mexican birth records in an attempt to explain the higher rates of adverse birth outcomes among the U.S.-born in Mexico compared to native-born women in Mexico. Table 4 provides a demographic and health care profile of the two groups of women using a larger number of educational and marital status categories and health care variables that are not comparable to the U.S. system. Consistent with our conclusions from Table 1, U.S.-born women in Mexico are slightly better educated and slightly less likely to be married or in a consensual union than Mexican-born women in Mexico, but the two groups are largely similar on these two characteristics.

**Table 4.** Demographic and Health Care Characteristics among 9,452,010 Births to U.S.-Born and Mexican-Born Women Delivering in Mexico, 2015–2019 (%).

	Mexican-Born	U.S.-Born
Education		
Elementary or less	20.0	18.0
Junior high school	39.8	37.2
High school	25.6	25.6
Bachelor’s	14.7	19.3
Marital status		
Married	37.2	34.7
Single	10.3	13.5
Consensual union	52.5	51.8
Nonmedical attendance	2.1	1.7
Type of medical insurance		
None	14.9	41.5
Public linked to work	30	18.5
Public linked to universal care	53.5	37.4
Other	1.5	2.6
Place of delivery		
Public linked to universal care	53.4	44.8
Public linked to work	22.9	12.1
Private	22.1	41.6
Other	1.6	1.5
Number of births	9,439,637	12,373

*Source:* Mexican birth records, 2015–2019.

The main differences between the two groups are seen in the type of medical insurance and the place of delivery. U.S.-born women in Mexico report a notable lack of social protection. A far greater share of U.S.-born women than Mexican-born women report having no health insurance (41.5% versus 14.9%). U.S.-born women are far less likely than Mexican-born mothers to have public insurance linked to formal employment (e.g., IMSS and ISSSTE) or universal public insurance (*Seguro Popular*). U.S.-born women are also less likely to have delivered in a public hospital linked to universal care or formal work. The largest group of U.S.-born women (41.6%) delivered in a private hospital, suggesting that many of them paid for the delivery out of pocket.

Table 5 shows the results of the linear regression analysis for low and very low birthweight and preterm birth. The model includes controls for the mother’s age, parity, education, and marital status and the number of prenatal visits, medical insurance, professional attendance, and place of delivery. As in the binational analysis, we still observe a (.010) higher probability of low birthweight and (.005) higher probability of very low birthweight among U.S.-born women compared to Mexican-born women, controlling for the more extensive set of variables. Consistent with their higher probability of low (and very low) birthweight, U.S.-born women also have a (.018) higher probability of preterm birth.

**Table 5.** Linear Probability Regression Coefficients of Adverse Birth Outcomes among 9,452,010 Births to U.S.-Born and Mexican-Born Women in Mexico, 2015–2019.

	Low Birthweight	Very Low Birthweight	Preterm Birth
	Coefficient (*SE*)	Coefficient (*SE*)	Coefficient (*SE*)
U.S. mothers (reference = Mexican-born mothers)	.010*** (.002)	.005*** (.001)	.018*** (.002)
Infant sex (male)	.003*** (.000)	.001*** (.000)	.007*** (.000)
Age (reference = 20–24)			
10–14	.023*** (.001)	.004*** (.004)	.030*** (.001)
15–19	.004*** (.000)	.000 (.000)	.006*** (.000)
25–29	.002*** (.000)	.001*** (.000)	.003*** (.000)
30–34	.009*** (.000)	.003*** (.000)	.013*** (.000)
35–39	.020*** (.000)	.005*** (.000)	.030*** (.000)
40–44	.035*** (.001)	.007*** (.000)	.049*** (.001)
45–49	.058*** (.002)	.009*** (.000)	.069*** (.003)
Parity (reference = first pregnancy)			
Second	–.008*** (.000)	–.001*** (.000)	–.003*** (.000)
Third or more	–.008*** (.000)	–.002*** (.000)	.003*** (.000)
Educational attainment (reference = elementary school or less)			
Junior high school	.003*** (.000)	.001*** (.000)	.005*** (.000)
High school	.004*** (.000)	.002*** (.000)	.008*** (.000)
Bachelor’s	.011*** (.000)	.003*** (.000)	.019*** (.000)
Marital status (reference = married)			
Single (no partner)	.004*** (.000)	.001*** (.000)	.002*** (.000)
Consensual union	.000 (.00)	.000 (.000)	–.003*** (.000)
Nondoctor attendance	–.015*** (.001)	–.002 (.000)	–.017*** (.001)
Medical insurance (reference = none)			
Public linked to work	–.002*** (.000)	–.000*** (.000)	–.001* (.000)
Public link to universal care	–.011*** (.000)	–.002*** (.000)	–.010*** (.000)
Other	.004*** (.001)	.001*** (.000)	.013*** (.001)
Place of delivery (reference = universal care)			
Public linked to work institution	.001** (.000)	.002*** (.000)	.012*** (.001)
Private institution	–.019*** (.000)	–.004*** (.000)	–.024*** (.000)
Other institution	–.026*** (.001)	–.004*** (.000)	–.029*** (.001)
Number of prenatal visits	–.004*** (.000)	–.001*** (.000)	–.004*** (.000)
Constant	.080*** (.000)	.012*** (.000)	.082*** (.001)
*R* ^2^	.0053	.0035	.0081
*F* statistic	1622.60	1084.04	2497.89
Number of observations	8,990,863	8,990,863	8,990,863

*Source:* Mexican birth records, 2015–2019.

* *p* < .05, **p < .01, ***p < .001.

## Discussion

In the first two decades of the twenty-first century, the population of U.S.-born children living in Mexico doubled in size to more than half a million as hundreds of thousands of Mexican immigrants returned to Mexico with their U.S.-born children (Masferrer et al. 2019; Masferrer and Pedroza 2021). This shift in migration flows signals a new era in Mexico-U.S. migration in which it is no longer sufficient to consider Mexican migrant health with a singular focus on immigrants in the United States (Escobar Latapí and Masferrer 2022). We build on a small body of innovative research combining Mexican and U.S. data sources to answer questions about immigrant health (Beltrán-Sánchez et al. 2016; Bostean 2013; Guendelman et al. 2015; McDonald et al. 2013; Riosmena et al. 2013), but we turn attention to immigrants in Mexico: the 0.5 generation. Our study provides the first examination of the health of the 0.5 generation by focusing on the health of their infants born in Mexico, newborns who have the right to both U.S. and Mexican citizenship.

In our binational analysis of Mexican and U.S. birth records from 2015 to 2019, we documented an immigrant health disadvantage in Mexico: Infants born to U.S.-born women in Mexico in this period had higher rates of low birthweight and very low birthweight than infants born to Mexican-born women in Mexico. The rate of low birthweight among births to U.S.-born mothers in Mexico was a full percentage point higher than the rate among births in Mexico to Mexican-born mothers, representing a 21% difference. This difference is greater than that observed between U.S. mothers with and without a high school degree and equivalent to the difference observed between U.S. mothers with and without some college (Martinson and Reichman 2016). If Mexican-born women in Mexico had the rate of low birthweight of U.S.-born women in Mexico, 94,396 more infants (= 9,439,637 × .01) would have been born with low birthweight.

Through the use of binational data, we contextualize the health of the 0.5 generation within the binational population of Mexican-origin migrants and nonmigrants giving birth in Mexico and the United States between 2015 and 2019. Our results suggest that infant health is stratified by country of mother’s birth, not migration status: Infants born to Mexican-born mothers during this period had lower rates of low birthweight than infants born to U.S.-born mothers regardless of where the mother delivered. In other words, we observe a health disadvantage common to U.S.-born mothers of Mexican origin, both those living in the United States and those living in Mexico. Binational data allow this common disadvantage of U.S.-born women of Mexican origin relative to Mexican-born women to be observed.

Our binational analysis also answers questions about the protective roles of education, marriage, and prenatal care and differences in these characteristics across groups of Mexican-origin women in Mexico and the United States. Both Mexican-born and U.S.-born women delivering in the United States have higher levels of educational attainment, formal marriage, and prenatal care usage than women delivering in Mexico. The predicted rates of low birthweight from regression models show that if women in the United States had similar levels of education, marriage, and prenatal care usage as women in Mexico, their rates of low birthweight would be significantly higher.

A large literature on the health of Mexican immigrants in the United States has documented the paradoxical finding that despite lower levels of education and access to prenatal care, the Mexican immigrant population has relatively good health outcomes in the United States, which could result from migrant selectivity both into and out of the United States (Arenas et al. 2015; Diaz et al. 2016; Fuentes-Afflick et al. 1999; Guendelman et al. 1999, 2015; Hummer et al. 2007; Markides and Coreil 1986; Riosmena et al. 2013, 2017). Binational data are necessary for assessing migrant selectivity. Similar to Guendelman et al. (2015), we do not find evidence of positive selection into immigration to the United States insofar as infants born to Mexican-born women in the United States have similar levels of low birthweight as infants born to Mexican-born women in Mexico. Unhealthy selection of immigrant parents into return migration may produce a less healthy population profile among the U.S.-born children who accompany them, but we did not find differences in the population health profiles of the 0.5 and the second+ generations, either. However, our results comparing groups of migrants and nonmigrants are only suggestive of selection. A more rigorous test of selectivity should observe people before and after migration and nonmigrants at similar time points.

Our results are consistent with a growing body of literature documenting the disadvantages faced by the 0.5 generation of U.S.-born children living in Mexico, a population now aging into adulthood. Studies document their educational delays and lags, poor educational outcomes, and low levels of health insurance coverage (Giorguli-Saucedo et al. 2021; Medina and Menjívar 2015; Sánchez Garcia and Hamann 2017; Vargas Valle and Ortiz Rangel 2019; Zúñiga and Hamann 2015, 2020). Our findings suggest that these disadvantages have health consequences for the offspring born to the 0.5 generation in Mexico at the very start of their lives. Given that low birthweight is strongly correlated with subsequent morbidity and developmental outcomes, our findings suggest a troubling health disadvantage that may persist beyond infancy (Boardman et al. 2002; Currie 2011; McCormick 1985).

A limitation of our study is that in our focus on the 2015 to 2019 period, we focus on births to a young population of U.S.-born people in Mexico. One-third of births among this group were to teenagers, and yet age-specific fertility rates were low among the U.S.-born at all childbearing ages. We suspect that the period captures a tempo distortion on fertility (i.e., low period fertility rates as a result of the postponement of childbearing for educational, family, or other purposes). The period of analysis may select an unrepresentative group of parents among the 0.5 generation: those who birthed early, who did not or could not delay childbearing. Future research should continue to document the health of this population through survey and registry data that identify place of birth and ideally, the migration trajectories of respondents.

Existing studies of the 0.5 generation emphasize the difficult conditions that return migrants and their children face (re)integrating into Mexican society after long periods of residence in the United States. As Zúñiga and Giorguli-Saucedo (2020) argue, the 0.5 generation in Mexico migrated in response to the “U.S. Great Expulsion”: intensive U.S. immigration enforcement in the interior of the country, which began in earnest after 9/11 and targeted long-term, settled immigrants, many with families (Schultheis and Ruiz Soto 2017). Returning to Mexico, these families encountered a country unprepared to accommodate the large-scale arrival of adults with foreign work credentials and their foreign-born children (Zúñiga and Giorguli-Saucedo 2020). Mexico’s government has since responded with efforts to assist returning migrants and their children (Masferrer and Pedroza 2021). In the United States, the reelection of Donald Trump in 2024 brought promises of mass deportation, which may result in a renewed level of migration to Mexico. President Trump also seeks to eliminate birthright citizenship, a right upheld by the 14th Amendment of the U.S. Constitution, a threat that targets many children of immigrants. As the political context and migration between Mexico and the United States evolve, continued attention to the health and well-being of binational citizens who have roots in both countries is essential.

## Notes

1. In this article, we use the term “women” to refer to all delivering people, although we recognize that not all people who give birth may identify as women.

2. ISSSTE stands for the *Instituto de Seguridad y Servicios Sociales de los Trabajadores del Estado*, the Institute for Security and Social Services for State Workers. PEMEX refers to *Petróleos Mexicanos*, Mexico’s state-owned oil and gas company. SEDENA is the *Secretaría de la Defensa Nacional*, Mexico’s army and air force. SEMAR is the *Secretaría de Marina*, Mexico’s navy. IMSS is the *Instituto Mexicano del Seguro Social*, or the Mexican Institute for Social Security.
